# Modification of Epoxides with Metallic Fillers—Mechanical Properties after Ageing in Aqueous Environments

**DOI:** 10.3390/ma16227181

**Published:** 2023-11-16

**Authors:** Anna Rudawska, Jakub Szabelski, Mariaenrica Frigione, Valentina Brunella

**Affiliations:** 1 Faculty of Mechanical Engineering, Lublin University of Technology, Nadbystrzycka 36, 20-618 Lublin, Poland; a.rudawska@pollub.pl; 2 Department of Innovation Engineering, University of Salento, Via Arnesano, 73100 Lecce, Italy; mariaenrica.frigione@unisalento.it; 3 Department of Chemistry, University of Torino, Via P. Giuria 7, 10125 Torino, Italy; valentina.brunella@unito.it

**Keywords:** epoxy, adhesives, modification, metallic fillers, ageing, aqueous environments, mechanical properties, compressive strength

## Abstract

The aim of this research was a comparative analysis of selected mechanical properties of epoxy compounds that were modified with metallic fillers and aged in aqueous environments. The tested epoxy compounds consisted of three components: styrene modified epoxy resin based on Bisphenol A, triethylenetetramine curing agent (resin/curing agent ratio of 100:10) and two types of metallic fillers in the form of particles: aluminum alloy (EN AW-2024–AlCu4Mg1) and tin-phosphor bronze (CuSn10P). Samples were subjected to ageing in 4 water environments: low-, medium- and high-mineralized natural water and in a sugar-containing solution for 1, 2 and 3 months. The epoxy samples were subjected to compressive strength tests in accordance with the ISO 604:2002 standard. It was observed that, among others, the compositions seasoned in low-mineralized water usually achieved the highest average compressive strength. As for filler type, using the bronze filler (CuSn10P) usually achieved the highest average compressive strength results.

## 1. Introduction

Modifications of polymeric materials are aimed at improving selected properties of the cured material [1]. They are most commonly carried out to modify the crosslinking reaction and influence the obtained network structure [2] in order to extend the life of the composition, reduce the exothermic effect of the crosslinking reaction [3] or accelerate the crosslinking process [4]. In addition, they can provide, for example, a reduction in manufacturing costs [5]. Their tasks are also to reduce the brittleness of the cured resin [6,7]; increase mechanical strength [8,9]; increase resistance to dynamic loads [10]; improve chemical [11], dielectric [12,13] and thermal resistance [14,15]; minimize flammability [16] and smokiness [17] and in general, expand the range of applications.

For polymer materials, there are several types of modifications: chemical modifications, physical modifications and physical–chemical modifications. Chemical modifications involve, for example, changing the type and amount of hardener to adjust the curing time, strength, chemical and low/high temperature resistance of the material [18,19,20,21]. In physical modifications of epoxy resins, the most commonly used agents include fillers, stabilizers, plasticizers and thinners. To expand the range of applications, special modifiers or other additives are used to change the properties of the substance. Typically, about 20% of the weight of artificial materials is simply modifying agents. With the help of some fillers, the fluidity of adhesives can be adjusted [22]. It is possible to increase the viscosity of the composition to the point that a paste is formed. When fillers are used in epoxy compounds, they reduce shrinkage and at the same time increase the strength of the joint (even doubling it). A distinction is made between fillers of the often-used type: (quartz sand or aluminum oxide, or slightly more expensive—silver flakes) and very expensive ones (such as boron fibers).

Adding reduced graphene oxide (RGO) up to 0.5 wt% increased the glass transition temperature, the modulus of elasticity and the strength of the epoxy adhesive and reduced the adhesive’s strain at the break at the ambient temperature, but the ultimate strain of RGO-reinforced adhesives decreased slightly [23]. Various types of biofillers [24,25,26,27], such as waste linseed cake, can also be used in small amounts to improve the value of the material’s storage modulus [28]. Biochar derived from wheat straw can be used to double the Young’s modulus and tensile strength, and biochar from miscanthus can be used to increase the elongation by 40% [6]. Other biochars are also used [29,30]: bamboo fibers [31], palm fibers [32], microcork particles [33], etc.

Aluminum oxide, silica and asbestos (inorganic fillers) increase strength and also contribute to heat resistance [14,34,35]. Fillers are also used to reduce or increase density. The introduction of a filler into the composition (by choosing the right type, as well as its granulation and pre-treatment) can also change chemical resistance and water absorption. By using silica, the rate of water vapor diffusion is reduced, while by using titanium white, the adsorption of solvent vapors is reduced.

However, the use of modifiers can lead to undesirable effects. The most important of these is increased viscosity of the composition, which makes it more difficult to process. In addition, problems such as poor adhesion, blistering, unevenness or discoloration on the surface and other potentially unfavorable hazards can occur.

The operating environment of polymeric materials has a significant impact on their strength and durability. Aging is associated with changes in the characteristics of the polymer material due to factors such as UV radiation [36,37,38], temperature and its variation [39,40,41,42,43], humidity/water environment [44,45,46,47,48,49,50,51], chemical agents [52,53,54,55] or simply long storage time [56]. Long-term exposure to atmospheric factors can lead to a reduction in their tensile, compressive and bending strength, etc. Some materials may become more brittle or react with a reduction in elasticity. In order to minimize the effects of the seasoning environment on the mechanical properties of polymeric materials, additives such as UV stabilizers and antioxidants, protective coatings and structural changes are used to minimize the material’s exposure to adverse conditions. It is then important to regularly test materials to monitor changes in mechanical properties. Thus, it seems important to study the behavior of epoxy materials with different types of fillers, seasoned in varying environments, as this comparison can provide important insights into the effect of seasoning time and type of operating environment on the strength of the target epoxy material, such as in [40].

Many construction solutions require materials with significant compression strength and adhesive properties, as they are intended as joining or sealing materials [57]. In many cases, the function of bonding and sealing is also combined with resistance to external factors, e.g., moisture or water (including rain). This situation may apply to the construction of building elements. Therefore, the aim of this work was to propose the preparation of modified epoxy materials, which are assumed to have greater strength compared to unmodified materials. Additionally, the compressive strength of such elements in various water environments and also in a sugar-containing environment was assessed. The aim was to obtain information that could be used when selecting materials for construction solutions.

In this paper, a comparative analysis of selected mechanical properties of epoxy compounds modified with metallic fillers (aluminum alloy and tin-phosphorus bronze) that were seasoned in aqueous environments was carried out. Five experimental environments were analyzed as aqueous environments: three aqueous environments with different degrees of mineralization (low-mineralized water, medium-mineralized water and high-mineralized water), a carbonated beverage with high sugar content and an ambient environment in which the seasoning process was not implemented—for comparison purposes. The experimental study provided information on the properties of modified epoxy compounds exposed to aqueous environments, which can be used in the design of adhesive joints and the development of adhesive technology.

## 2. Materials and Methods

### 2.1. Epoxy Compound

The following components were used in epoxy compounds: a styrene-modified epoxy resin based on a bisphenol A (Epidian 53) with an epoxide number of min. 0.41 mol/100 g (DGEBA and styrene) and a triethylenetetramine curing agent (Z-1 trade name) with an amine number of min. 1100 mg KOH/g. Both main components of the epoxy composition were produced by the “Organika-Sarzyna” Chemical Plant in Nowa Sarzyna, Poland.

The selected properties of the epoxy resin and curing agent are shown in Table 1.

A triethylenetetramine curing agent (amine type) used most often for compounds with low molecular weight epoxy resins. Table 2 shows the properties of amine curing agent.

Two types of particulate materials were used as metallic fillers: aluminum alloy (EN AW-2024 AlCu4Mg1) and tin-phosphorus bronze (CuSn10P).

Aluminum alloy (EN AW-2024) is a lightweight metal with low oxidation resistance and is difficult to weld. It is characterized by a large amount of copper and high fatigue strength. These materials find important applications in the automotive and aerospace industries, as well as in the construction industry—if there is no risk of corrosion. The physical properties of the aluminum alloy used are shown in Table 3. The particle size of the aluminum alloy was 0.08 ÷ 0.10 mm.

Tin-phosphorus bronze (CuSn10P) is an alloy of copper with tin and an admixture of phosphorus. The copper content is approximately 90%, the tin content is 1–9% and the phosphorus content is 0.1–0.3%. It is typically used in the manufacture of components for heavy-duty and high-speed operation, e.g., corrosion-prone bearings; machine parts including shafts, bushings and other components; and chemical fittings. Since tin-phosphorus bronze is characterized by very good self-lubricating properties, it is used for the manufacture of poorly lubricated components, such as those that are difficult to access and exposed to long intervals of operation without maintenance. The particle size of the tin-phosphorus bronze was 0.05 ÷ 0.10 mm. The basic physical properties of bronze are shown in Table 4.

**Figure 1:**
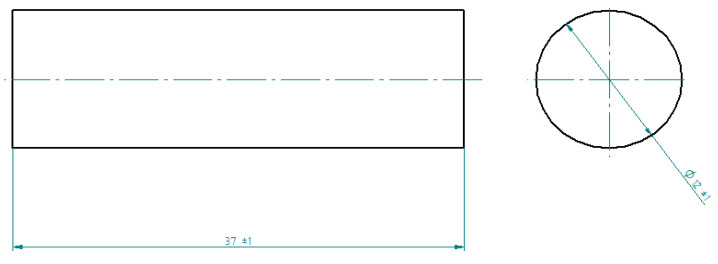
The cylindrical epoxy compound sample.

The main components of the epoxy composition, the resin and curing agent were mixed, taking into account the appropriate stoichiometric ratio recommended for these specific components. For this resin and curing agent, the stoichiometric resin:curing agent ratio is 100:10. The compounds adopted in the research plan (Table 5) were prepared by mixing the ingredients for 2 min using a table-top drill at 730 rpm and then placed in molds with walls coated with release agent. The epoxy compound samples used in the study were produced in a cylindrical mold of diameter ⌀12 ± 1 mm and length 37 ± 1 mm (Figure 1).

The following conditions were used during curing: temperature: 20 ± 2 °C, humidity: 26 ± 1% and curing time: 7 days. A total of 78 samples of each epoxy compound were made.

### 2.2. Test Environment

Five test environments were used for the study. The aqueous environments encompassed water classified into three different groups according to the current Regulation of the Polish Minister of Health of 31 March 2011 on natural mineral waters, spring waters and table waters, Annex 5—“Criteria for chemical classification used in the labelling of natural mineral waters”. These were: low-mineralized water, medium-mineralized water and high-mineralized water (Table 6). A carbonated beverage with a high sugar content was used as a fourth environment. In addition, some samples were analyzed in ambient environments (without a seasoning process). Table 7 shows the labelled operating environments.

The water types used for testing are extracted from underground and show purity and the absence of chemical as well as bacterial contamination. They all have a one-year expiry date set by the manufacturers (LMW: Polska Woda, Ozorków, Poland, MMW: Mineral, Gorzanów, Poland, HMW: Cechini, Muszyna, Poland) and specific storage conditions (dark and cool places). The ingredients of the sweet drink used in the study are water, glucose-fructose syrup, sugar, color, ammonia-sulphite caramel, phosphoric acid, acidity regulator and flavors. It is also unlike the waters presented above in being carbonated. This drink, according to the manufacturer’s recommendation, should be stored in shaded and cool areas.

### 2.3. Experimental Research Methodology

The modified epoxy samples were cured for 7 days at 20 ± 2 °C and humidity: 26 ± 1%. Series of 6 cured samples were placed in varying operating environments including one series in ambient conditions. The seasoning times for each series were: 1, 2 and 3 months. The seasoning conditions (shaded, cool location) were identical for all samples.

After fabrication, the specimens of the cured epoxy compounds were milled to remove surface irregularities, which was necessary for proper strength testing. Compressive strength tests were carried out on a ZWICK/ROELL Z150 machine (Zwick Roel, Ulm, Germany) according to the ISO 604:2002 Plastics—Determination of compressive properties [58], with constant parameters for all the specimens. Due to the fact that for many construction materials, e.g., those used in the building industry, the basic mechanical property is compressive strength, an attempt was made to determine this type of strength to compressive load.

## 3. Results

### 3.1. Variability of Obtained Results

The coefficient of variation (COV), as a measure of the variability of the data in a series of samples, describes the degree of dispersion or variability of the data (standard deviation SD) in relation to its mean value (MV). It is expressed by the Formula (1):

COV = (SD/MV) × 100%(1)

A coefficient of variation of less than 15% is estimated to define a series with low variability, i.e., those in which the individual values are relatively close to the mean value and there are no large discrepancies between them.

Table 8 shows the summary results of the distribution of results in the individual series, depending on the material tested, the environment and the seasoning time.

It can be observed that all the series studied had a very low variability, somewhere between 1 and 9%. At the same time, there is a clear difference between the variability of the results of the individual series depending on the tested epoxy material. The E53/Z-1/Al showed almost twice the average variability of the unmodified epoxy compound (E53/Z-1), while E53/Z-1/Br showed more than twice as much variability. Still, the results of the dispersions make it possible to describe all series as having low variability. This indicates, on the one hand, a high consistency of results and, indirectly, also a correctly selected minimum number of samples in each series.

A more detailed analysis of the dependence of the degree of dispersion of the results in relation to the seasoning time and the seasoning environment did not show similar relationships.

### 3.2. Comparative Analysis of Test Results within the Same Seasoning Time

#### 3.2.1. Test Results for Unseasoned Epoxy Compound (O)

**Figure 2:**
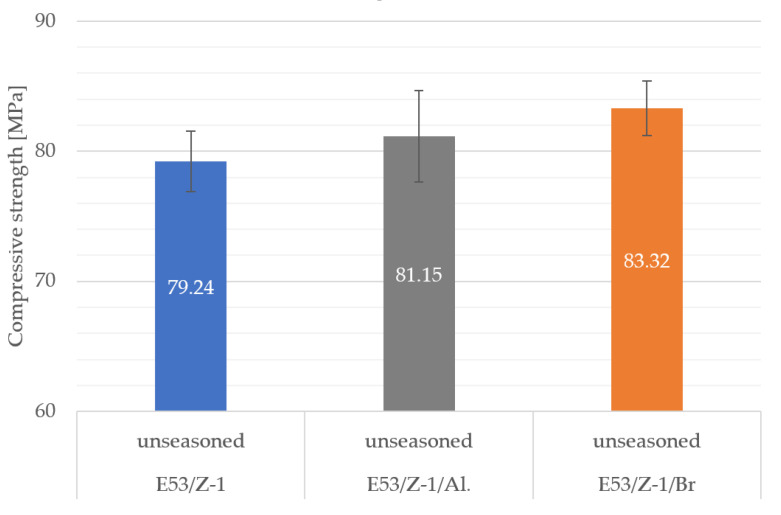
Comparative analysis of strength results of unseasoned epoxy compound in an aqueous environment.

The results of the unseasoned epoxy compound samples that were not seasoned in aqueous environments were analyzed. Figure 2 shows the strength results of the samples of the epoxy compound tested: E53/Z-1, E53/Z-1/Al, E53/Z-1/Br.

The samples with the epoxy compound Epidian 53 with Z-1 curing agent and bronze filler (E53/Z-1/Br) clearly showed the highest average compressive strength of 83.32 MPa. The difference between the average strength of the strongest and weakest samples was only 5%. The scatter in the results obtained with the E53/Z-1/Al epoxy compound was noticeably greater than with the other two epoxy compounds.

#### 3.2.2. Test Results for Epoxy Compounds Seasoned in Different Environments

Table 9 shows a summary of the compressive strength of epoxy compounds results obtained, depending on the environment and seasoning time.

When analyzing the average results obtained after 1 month of seasoning (Table 9), the following patterns were noted. Samples E53/Z-1/Al had the lowest average strength (79.30 MPa), while samples E53/Z-1/Br had the highest (87.65 MPa). The difference in average strength between the two is 8.35 MPa, representing 9.53%. Both values were obtained in a medium mineralized water environment. In high-mineralized water, the difference between the smallest (E53/Z-1/Al (80.67 MPa) and the largest mean (E53/Z-1 84.68 MPa) is only 4 MPa (4.74%), while in low-mineralized water and the CO_2_-saturated high-sugar beverage, the differences were only about half the size.

The individual compositions responded differently to the seasoning environment. E53/Z-1 between the highest (in high-mineralized water 84.68 MPa) and lowest average strength (for the high-sugar beverage 79.90 MPa) achieved a difference of 4.78 MPa (5.65%). For the E53/Z-1/Al composition, the difference was 3.73% (max. in low-mineralized water 82.37 MPa, min. in medium-mineralized water 79.30 MPa) and E53/Z-1/Br 5.93 MPa, i.e., 6.75% (min. in high-sugar beverage 81.72 MPa, max. in medium-mineralized water 87.65 MPa).

Looking in detail at the results obtained from the samples after 2 months of seasoning, it can be seen that the highest average compressive strength among the compositions tested was obtained for samples seasoned in highly mineralized water in the case of the E53/Z-1/Br composition (86.08 MPa), while the lowest was obtained for the E53/Z-1 composition seasoned in a high-sugar beverage (77.6 MPa). The difference between these extreme averages was 8.48 MPa, which represents 9.8%. The behavior of the tested compositions in low-mineralized water and the high-sugar beverage was quite similar, as can be seen by only a 3.2% and 4.23% difference between the largest and smallest results obtained. However, in the case of the high-mineralized water, it was 6.75%, and in the medium-mineralized water, it was 8.3% (78.72 MPa–E53/Z-1/Al vs. 85.84 MPa E53/Z-1/Br).

Analyzing the results obtained after 3 months of seasoning, the following observations can be noted. There is a clear difference of up to 16.5% between the most and least durable composition (E53/Z-1 in high sugar beverage 75.63 MPa vs. E53/Z-1/Br in low mineralized water 90.58 MPa). Within the individual seasoning environments, the differences were significantly smaller: 8.3% in the low-mineralized water, 6.6% in the high-sugar beverage, 5.4% in the medium-mineralized water and 2.6% in the high-mineralized water.

### 3.3. Comparative Analysis of Research Results within a Common Seasoning Environment

**Figure 3:**
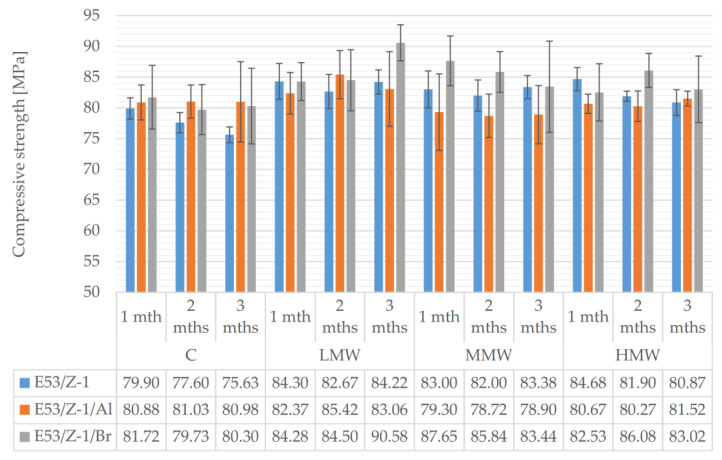
Comparative analysis of compressive strength results of epoxy compounds specimens grouped by seasoning medium.

An attempt to summarize all the results obtained for the compressive strength of the epoxy material samples grouped according to the type of environment and seasoning time is shown in Figure 3.

Analyzing the results obtained after seasoning in low-mineralized water, it was noted that, irrespective of the seasoning time, the difference between the highest (E53/Z-1/Br for 3 months: 90.58 MPa) and lowest (E53/Z-1/Al for 1 month: 82.37 MPa) average compressive strength obtained was 8.21 MPa (9.1%). Within the individual epoxy compounds, in fact, a notable difference was only observed between the samples made from E53/Z-1/Br seasoned for 1 and 3 months: namely, the 3-month samples proved to be 6.3 MPa stronger (7%). The other two compositions had differences between minimum and maximum average compressive strengths in the range of 2–3%.

In the case of medium mineralized water, the higher average strength of the epoxy samples filled with tin-phosphorus bronze was clearly seen, especially at the beginning of the seasoning. The difference between the least durable material (E53/Z-1/Al seasoned 2 months—78.72 MPa) and the most durable (E53/Z-1/Br seasoned 1 month—87.65 MPa) exceeded 10% (8.93 MPa). Comparing the average values obtained for the different materials, it is evident that the influence of the seasoning of the undoped material (E53/Z-1) and aluminum-doped material (E53/Z-1/Al) is very small: 0.7–1.2%. Only the samples in which bronze (E53/Z-1/Br) was used achieved a decrease in average compressive strength of 4.2 MPa (approx. 5%).

Samples aged in highly mineralized water showed average strength changes with seasoning time. Globally, a 6.7% difference was registered between the strongest and weakest samples—the average strength of the bronze-doped epoxy was 5.8 MPa higher than that of the aluminum-filled epoxy. It was the E53/Z-1/Al samples that were the most stable during seasoning, showing only a 1.5% difference between the seasoning series. A 4.1–4.5% difference (3.55 MPa–3.81 MPa) was observed between the undoped and bronze-doped samples.

Using aluminum filler again resulted in the greatest stability of the average compressive strength results when the material was seasoned in a CO_2_-saturated, high-sugar beverage. These values were essentially unchanged over time (0.1% difference). A slightly larger change was registered for epoxy with bronze (E53/Z-1/Br)—2.4%. In contrast, for the unmodified specimens, degradation progressing with seasoning was observed, changing by 4.3 MPa (5.3%). Comparing all materials with each other, the greatest difference was observed between the unmodified samples (75.63 MPa at month 3 of seasoning) and the modified bronze, which reached an average strength of 91.72 MPa after one month of seasoning. This gives a 7.5% difference between the averages.

### 3.4. Changes in Average Compressive Strength of Individual Epoxy Compounds

**Figure 4:**
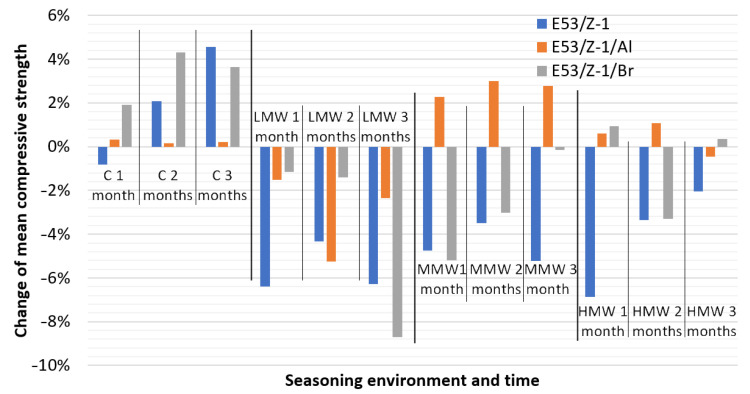
Change in mean compressive strength of tested samples of epoxy compounds.

Figure 4 shows the change in compressive strength compared to the unseasoned material specimens (Figure 2). A detailed analysis of the strength changes as a function of seasoning time and environment is presented for the individual compositions later in this paper.

#### 3.4.1. Unmodified Epoxy Compound E53/Z-1

The results of the compressive strength tests were analyzed according to the type of epoxy compound—E53/Z-1, which was seasoned in four environments for one, two and three months, and epoxy compounds that were not seasoned. The samples that were not seasoned achieved an average compressive strength of 79.24 MPa. The seasoned epoxy compound showed little change in average strength in two cases: when seasoned in low- and medium-mineralized water. In highly mineralized water and the Cola beverage, a progressive decrease in average compressive strength of up to approximately 5% was recorded with seasoning time. When analyzing the differences between the various compositional behaviors in the seasoning environments studied, it is worth noting that the epoxy compound showed a particularly large deterioration in compressive strength during seasoning in the carbonated high-sugar beverage, with the average values in this group being noticeably lower than in the mineralized water groups.

#### 3.4.2. Epoxy Compound Filled with Aluminum E53/Z-1/Al

The aluminum-doped composition, as noted above, proved to be quite stable and resistant to seasoning in the analyzed environments. In case of low-mineralized water—the difference in average compressive strength during seasoning did not exceed 3.6%. Water with a higher mineral content caused even less of a change in average strength: medium—0.5%, high-mineralized—1.5%. Similarly, a carbonated beverage with high sugar content registered a 0.2% change. Regarding the overall effect of the seasoning environment on the average compressive strength value, it is clear that lower values were obtained in the medium mineralized water compared to the other waters and the beverage. Meanwhile, the highest values were obtained in the group of samples seasoned in low mineralized water. However, it is difficult to draw detailed conclusions on this basis, as precisely these two groups (samples seasoned in low- and medium-mineralized water) were characterized by clearly higher standard deviation values.

#### 3.4.3. Epoxy Compound with Tin-Phosphor Bronze E53/Z-1/Br

Analyzing the results obtained, it can be seen that quite large differences were registered within the samples seasoned in different environments. Between the least and most durable variants, the difference reached 12%. Clearly, the lowest values of average compressive strength were obtained with the epoxy compound in the environment of a carbonated beverage with high sugar content. And in this environment, the results obtained were the most consistent, with a min-max difference of 2.4%. The less mineralized the water used for seasoning, the greater the differences within the group. That is, for highly mineralized water, 3.5 MPa (4.1%) was recorded between the lowest and highest mean strength values, for medium mineralized water it was 4.2 MPa (4.8%) and low mineralized water showed a difference of 6.3 MPa (7%).

## 4. Discussion

The studied epoxy compounds were based on Epidian 53 epoxy resin, Z-1 curing agent and fillers in the form of particles of aluminum alloy (EN AW-2024) and tin-phosphorus bronze (CuSn10P). The cured compounds were seasoned in four experimental environments—low-, medium-, and high-mineralized water and also in a carbonated high-sugar beverage for periods of one, two and three months.

The application of fillers to polymeric materials, in this case epoxy, was carried out in order to obtain better properties of the cross-linked material. It may happen that for the price of improving selected characteristics, others may deteriorate. The selection of the right filler and its amount for use in the epoxy is therefore crucial to achieving the desired goals. Hence, experimental studies on the effect of selected fillers on the various characteristics of specific epoxy compounds are needed.

Epoxy materials can be degraded by water sorption. The mechanism of this interaction has been analyzed in many works. When testing the effect of water on epoxy coatings, using infrared spectroscopy and dielectric measurements, the appearance of hydrogen bonds was observed during hydrothermal aging [59]. The analysis of water sorption of various epoxy compounds by gravimetric measurements and positron annihilation lifetime spectroscopy (PALS), on the other hand, showed that, regardless of the test temperature, the free volume fraction is not a decisive factor regulating the equilibrium water content, while the polarity of the system is an essential factor [60].

The degree of saline water absorption by epoxy coatings and the swelling of these coatings, regardless of the solution temperature, were lower for coatings with physical aging than for coatings without aging. Thermodynamic analysis of the diffusion parameters showed that thickening of the polymer matrix induced by physical aging and, consequently, a higher number of interactions between the polar groups of polymer chains, which reduce the number of polar groups for water diffusion, are the main factors regulating the process of water absorption [61]. Similar results were obtained for adhesive joints made with aged epoxy adhesives. Under cyclic aging conditions, the moisture diffusion constant and maximum moisture content increase with aging cycles and mechanical properties such as elastic modulus and tensile strength decrease as the number of aging cycles increases [62].

The progress of the crosslinking process is also important. It has been shown that depending on the curing temperature of DGEBA/TETA compounds, despite the achievement of complete crosslinking, different curing conditions affect the free volume and activation volume associated with viscoelastic properties. Compounds cured at temperatures below Tg exhibit higher mechanical properties and are less susceptible to water ingress than systems cured at temperatures above Tg [63].

In the present research, degradation of the average compressive strength of epoxy compound samples with metallic fillers was recorded in almost all of the analyzed cases of aging the epoxy material in a mineral water environment. Only the epoxy compound filled with aluminum resisted deterioration of properties when it was seasoned in medium and highly mineralized water. On the other hand, ageing in the environment of a carbonated beverage with high sugar content did not lead to such a clear decrease in the compressive strength properties.

To conclude, when ageing the epoxy compounds in low-mineralized water, the results show a slight improvement in strength properties. This may be due to the presence of fewer mineral components. These components generally have a negative effect on the strength of epoxy compounds.

The right choice of filler type affects the mechanical strength of the epoxy material. The study showed that bronze was the most favorable filler. The results may have indicated a higher strength due to a better distribution of bronze particles in the cylindrical molds used to shape the samples from the tested composition.

The topic of modification of epoxy compounds with metallic fillers is extensive and needs to be further explored. Conducting further research will allow us to continuously improve and gain more knowledge about the modification of epoxy compounds with metallic fillers.

## 5. Summary

This study was conducted to test the compressive strength of epoxy compounds with metallic fillers. Analysis of the results established that the environment and seasoning time affect the compressive strength results. The following conclusions can be drawn:

- The epoxy compound that has been seasoned in low-mineralized water usually obtains the highest strength results: the highest average compressive strength was achieved by E53/Z-1/Br samples, which were seasoned in low-mineralized water for a period of three months; the strength of this epoxy compound is 90.58 MPa,
- The seasoning environment—in the form of a carbonated beverage—is not particularly beneficial to obtaining high-strength results; it was during the seasoning of the epoxy compound in this environment that the lowest strength results usually occurred,
- The epoxy compound with bronze filler (CuSn10P) tended to obtain the highest compressive strength results,
- Samples of epoxy compounds without filler usually achieved the lowest strength results: the lowest average strength was achieved by the E53/Z-1 epoxy compound, which was seasoned for a period of three months in a carbonated high-sugar beverage and reached an average of only 75.63 MPa.

The practical recommendations resulting from the research include:

- In general, it can be seen that the content of minerals in the aqueous environment influences the mechanical properties of epoxy materials, and a smaller amount of minerals is recommended,
- A sugar-containing environment negatively affects the properties of epoxy materials; therefore, exposing such materials to this type of environment should be avoided,
- During modification, the type of modifying agent is important, which is why it is necessary to analyze the use of epoxy materials in specific construction solutions along with the required strength properties.

## Figures and Tables

**Table 1:** Properties of epoxy resin.

Properties	Value
Flash point	75 [°C]
Boiling point	1410 [°C]
Density at 20 °C	1.11–1.15 g/cm^3^
Viscosity at 20 °C	900–1500 mPa·s
Epoxy number	0.41 mol/100 g
Gelation time with Z-1 curing agent	60 min

**Table 2:** Properties of triethylenetetramine curing agent.

Properties	Value
Amine number	min. 1100 mgKOH/g
Boiling point at 13 hPa	143 °C
Boiling point at 66.5 hPa	183 °C
Viscosity at 25 °C	20–30 mPa·s
Density at 25 °C	0.98 g/cm^3^

**Table 3:** Physical properties of aluminum alloy (EN AW-2024 AlCu4Mg1).

Properties	Value
Density	2.78 g/cm^3^
Tensile strength Rm	360–435 MPa
Hardness	104–123 HB

**Table 4:** Physical properties of tin-phosphorus bronze (CuSn10P).

Properties	Value
Density	7.5–9.3 g/cm^3^
Casting shrinkage	1.5–2.5%
Melting point	940–1084 °C

**Table 5:** Epoxy compounds.

Resin and Curing Agent	Filler	Denotation
DGEBA + styrene (Epidian 53) (100 g) and TETA (Z-1) (10 g) (10:1)	-	E53/Z-1
	1% aluminum alloy AW-2024 AlCu4Mg1	E53/Z-1/Al
	1% tin CuSn10P	E53/Z-1/Br

**Table 6:** Aquatic environment parameters.

Aqueous Environments	Low-Mineralized Water (LMW)	Medium-Mineralized Water (MMW)	High-Mineralized Water (HMW)
Trade name	Spring water Aleksandria	Natural mineral water from the Sudety	CECHINI MUSZYNA
Manufacturer	Polska Woda, Ozorków, Poland	Mineral, Gorzanów, Poland	Cechini, Muszyna, Poland
Mineral quantities	50–500 mg/L	500–1500 mg/L	1500–4000 mg/L
CATIONS			
Calcium	52.1 mg/L	198.0 mg/L	325.8 mg/L
Magnesium	7.3 mg/L	18.4 mg/L	61.6 mg/L
Sodium	2.5 mg/L	94.3 mg/L	33.2 mg/L
Potassium	0.86 mg/L	7.3 mg/L	3.7 mg/L
ANIONS			
Bicarbonate	143.4 mg/L	812.0 mg/L	1314.0 mg/L
Sulphate	27.8 mg/L	54.0 mg/L	16.5 mg/L
Chlorine	10.3 mg/L	40.0 mg/L	8.9 mg/L
Fluorine	0.13 mg/L	0.49 mg/L	0.11 mg/L

**Table 7:** Labeling of ageing environments.

Type of Environment	Label
Ambient conditions	O
Low-mineralized water	LMW
Medium-mineralized water	MMW
High-mineralized water	HMW
Carbonated high sugar beverage	C

**Table 8:** Coefficient of variation of sample series.

Environment	Seasoning Time	Epoxy Compound		
		E53/Z-1	E53/Z-1/Al	E53/Z-1/Br
C	1 month	2.16%	3.52%	6.34%
	2 months	2.13%	3.33%	5.08%
	3 months	1.69%	8.06%	7.66%
LMW	1 month	3.47%	4.10%	3.65%
	2 months	3.36%	4.57%	5.86%
	3 months	2.32%	7.32%	3.23%
MMW	1 month	3.61%	7.85%	4.62%
	2 months	3.11%	4.48%	3.85%
	3 months	2.25%	5.99%	8.90%
HMW	1 month	2.21%	1.95%	5.64%
	2 months	1.01%	3.09%	3.23%
	3 months	2.60%	1.47%	6.51%
	Mean COV	2.49%	4.65%	5.38%

**Table 9:** Compressive strength (mean values) of epoxy compounds.

Environment	Seasoning Time	Epoxy Compound		
		E53/Z-1	E53/Z-1/Al	E53/Z-1/Br
C	1 month	79.90	80.88	81.72
	2 months	77.60	81.03	79.73
	3 months	75.63	80.98	80.30
LMW	1 month	84.30	82.37	84.28
	2 months	82.67	85.42	84.50
	3 months	84.22	83.06	90.58
MMW	1 month	83.00	79.30	87.65
	2 months	82.00	78.72	85.84
	3 months	83.38	79.90	83.44
HMW	1 month	84.68	80.67	82.53
	2 months	81.90	80.27	86.08
	3 months	80.87	81.52	83.02

## Data Availability

Data are contained within the article.
